# Mucocutaneous Adverse Events in Patients With Cancer Treated with the Highly Selective RET Kinase Inhibitor Selpercatinib (LOXO-292)

**DOI:** 10.1016/j.jtocrr.2025.100792

**Published:** 2025-01-07

**Authors:** Rachel E. Reingold, Rose Parisi, Guilherme Harada, Andrea P. Moy, George Dranitsaris, Jasmine H. Francis, Julia Canestraro, Julia A. Lester, Lauren A. Kaplanis, Dazhi Liu, Mario E. Lacouture, Alexander Drilon

**Affiliations:** a Dermatology Service, Department of Medicine, Memorial Sloan Kettering Cancer Center, New York, New York; b Department of Dermatology, Weill Cornell Medical College, New York, New York; c Thoracic Oncology Service, Division of Solid Tumor Oncology, Department of Medicine, Memorial Sloan Kettering Cancer Center, New York, New York; d Early Drug Development Service, Division of Solid Tumor Oncology, Department of Medicine, Memorial Sloan Kettering Cancer Center, New York, New York; e Dermatopathology Service, Department of Pathology, Memorial Sloan Kettering Cancer Center, New York, New York; f Department of Public Health, Falk College, Syracuse University, Syracuse, New York; g Ophthalmic Oncology Service, Department of Medicine, Memorial Sloan Kettering Cancer Center, New York, New York; h Department of Medicine, Weill Cornell Medical College, New York, New York

**Keywords:** Selpercatinib, Mucocutaneous adverse event, RET fusion, RET mutation

## Abstract

**Introduction:**

Selective RET inhibitors are approved for the treatment of RET-dependent cancers. A comprehensive characterization of mucocutaneous adverse events (MAEs) has not been performed; therefore, we characterized MAEs associated with the selective RET inhibitor, selpercatinib.

**Methods:**

We assessed 133 patients with *RET-*altered cancers treated with selpercatinib. The type, grade, cumulative incidence, and time to onset of MAEs were determined. Therapy interruptions, clinicopathologic findings, and management were described. Laboratory values were compared between patients with and without MAEs.

**Results:**

A total of 73 patients with mostly NSCLC (n = 46, 63%), medullary thyroid (n = 19, 26%), and papillary thyroid (n = 6, 8%) cancers had 126 predominantly grade 1/2 (n = 124, 98%) MAEs, with 48% reporting greater than one MAE. Xerostomia (n = 49, 37%), rash (n = 24, 18%), periorbital edema (n = 16, 12%), and xerosis (n = 12, 9%) were the most common MAEs. The yearly cumulative incidence of all-grade MAEs was 55%, with a median time to onset of 57 (interquartile range: 15–166) days after initiation. Those with MAEs had a significantly higher percentage of lymphocytes (mean = 21.8, SD = 11.3, *p* = 0.005) compared with those without MAEs (16.9, SD = 10.0) and elevated immunoglobulin E (mean = 275, SD = 294.5 IU/mL). There were 18 (14%) MAE-related therapy interruptions, including the following: three (2%) rechallenged with dose maintained, 10 (7%) with a 50% dose reduction, 5 (4%) with a 25% dose reduction, and no drug discontinuations. A treatment algorithm was created for the most common MAEs: xerostomia managed with saliva and lubricants; mucositis with steroid rinses; rashes with topical steroids with or without topical ammonium lactate; periorbital edema with cold or caffeine compresses; and xerosis and pruritus with emollients.

**Conclusions:**

Selective RET inhibition is associated with a unique MAE profile. Early recognition and management of MAEs may improve quality of life, minimize interruptions, and maximize therapeutic benefit.

## Introduction

*RET* fusions and mutations exist in a subset of patients with NSCLC, and medullary and nonmedullary thyroid carcinomas.^1^ Selpercatinib (LOXO-292) is a highly selective RET kinase inhibitor with efficacy against *RET*-altered cancers, has the unique ability to penetrate the central nervous system, has minimal off-target effects, and has outperformed trials of multikinase inhibitors.^2^ As such, selpercatinib has been approved for the treatment of metastatic *RET* fusion–positive cancers of any type, and advanced or metastatic *RET*-mutant medullary thyroid cancers.^3^

The phase 1/2 LIBRETTO-001 trial of selpercatinib, including 531 patients with *RET*-altered cancers, reported mucocutaneous adverse events (MAEs) such as xerostomia (33%), edema (15%), and rash (12%). Although only 2% of patients had drug discontinuation, drug hypersensitivity reaction was a leading cause.^2^^,^^4^

The occurrence and improper management of MAEs with targeted cancer therapies have proven to negatively impact patients’ health-related quality of life, which may affect adherence to therapy.^5^^,^^6^

To date, selpercatinib-related MAEs have not been extensively assessed. On the basis of clinical trial success, we expect to see an increase in selpercatinib use for *RET*-altered neoplasms. Proper identification and management of selpercatinib-related MAEs may help to maximize therapeutic benefits and maintain quality of life. We aim to calculate the yearly cumulative incidence of MAEs after selpercatinib initiation in those with RET-altered cancers. In addition, we sought to characterize the clinicopathologic phenotype of selpercatinib-associated MAEs and present the management strategies used in a single-center cohort.

## Materials and Methods

We retrospectively assessed 133 patients with *RET*-altered cancer treated with selpercatinib from May 2017 to March 2021 at a single tertiary academic hospital, Memorial Sloan Kettering Cancer Center, New York, New York under an institutional review board–approved protocol (#16-458). Informed consent was waived given the retrospective review and because of the deidentification of clinical data and records, posing minimal risk to participants. Patients who followed up past March 2021 were censored. Patients with a history of selpercatinib therapy were identified using the institutional information systems service (Dataline). Patient demographics, cancer diagnosis, selpercatinib therapy data (initiation, dose, interruptions), MAE clinical features, management, histopathologic assessments, and laboratory values were extracted from the electronic medical record.

The primary outcome measure was calculating the yearly cumulative incidence of MAE after selpercatinib initiation. Secondary outcome measures included describing the MAE time to onset, therapy interruptions, clinical presentation, management, histopathologic features, and relevant laboratory values. All adverse events (AEs) were graded according to Common Terminology Criteria for AEs version 4.03 and version 5.0.^7^ Differing from the Common Terminology Criteria for AEs categorization of AEs, in the current study, AEs were considered to be mucocutaneous by virtue of anatomical location. This included all AEs related to mucosal (including lips, oropharynx, genitals, and eyes) and cutaneous surfaces (including edema, rash, or altered sensation of the skin like pruritus, pain, or neuropathy). Relatedness to selpercatinib was determined by clinical notes and adverse reaction panel documentation.

Clinically indicated biopsies from MAE onset were reviewed and available slides were retrospectively reassessed by a dermatopathologist to systematically evaluate histopathologic features. Laboratory values of interest included complete blood count with differential, comprehensive metabolic panel, and immunoglobulin E (IgE). For those with MAEs, laboratory values that were collected for clinical purposes were reviewed and recorded within more or less 14 days of MAE onset, meaning those with more than one MAE may have had multiple sets of laboratory values. Comparative laboratory values from no-MAE patients were collected within more or less 14 days of the calculated mean time to MAE onset (97 d). Those without values within this time range were excluded from the analysis.

Descriptive statistics were used to report demographics, yearly cumulative incidence of MAE, therapy interruption, and clinicopathologic characteristics. The Kaplan-Meier method was used for a time-to-event analysis of MAE onset for all-grade MAEs and the most often occurring MAE phenotypes after selpercatinib initiation. All time-to-event outcomes were reported as medians with the interquartile range (IQR). Post hoc analyses used logistic regression models to estimate odds ratios to measure the association between MAE incidence and patient demographic and clinical characteristics. To determine whether laboratory values differed in the MAE versus no-MAE group, an unpaired, two-sample *t* test with equal variances was used. All analyses were considered significant when the *p* value was less than 0.05 and there was no adjustment for multiplicity. Statistical data analyses were performed using Stata V16.0 (Stat Corp., College Station, TX).

## Results

### Study Population

A total of 133 patients including adults (n = 131, 98%) with a median age of 63 (range: 22–92) years and pediatric patients (n = 2, 2%) with a median of 10 (range: 2–17) years old were treated with single-agent selpercatinib for *RET* fusion (n = 96, 72%) or *RET* mutation (n = 37, 28%) positive cancers (Table 1). The population was 72% (n = 96) White and 52% (n = 69) were of male sex. Common primary cancer diagnoses included 59% (n = 78) with NSCLC, 27% (n = 36) with medullary thyroid cancer, and 9% (n = 12) with papillary thyroid cancer. Only 14% (n = 18) of patients received immunotherapy within 6 months of selpercatinib initiation. A total of 38 (29%) out of 133 patients died at a median of 396 (range: 7–1050) days after selpercatinib initiation. Most patients were treated with selpercatinib at 160 mg twice daily (n = 106, 79%); the remaining patients were put on a dose escalation scheme.

**Table 1:** Patient and Clinical Characteristics

Characteristics	With MAE (n = 73)	Without MAE (n = 60)	All (N = 133)
Age, y			
Median (range)	62 (17–88)	64 (2–92)	63 (2–92)
Sex, n (%)			
Male	35 (48)	34 (57)	69 (52)
Female	38 (52)	26 (43)	64 (48)
Race, n (%)			
White	56 (77)	40 (67)	96 (72)
Asian	8 (11)	11 (18)	19 (14)
Black	3 (4)	4 (7)	7 (5)
Native American/Alaskan Native	0 (0)	2 (3)	2 (2)
Other	6 (8)	3 (5)	9 (7)
Primary cancer diagnosis, n (%)			
NSCLC	46 (63)	32 (53)	78 (59)
Medullary thyroid cancer	19 (26)	17 (28)	36 (27)
Papillary thyroid cancer	6 (8)	6 (10)	12 (9)
Other^a^	2 (3)	5 (8)	7 (5)
*RET* alterations, n (%)			
*RET* mutation	20 (27)	17 (28)	37 (28)
*RET* fusion	53 (73)	43 (72)	96 (72)
Concurrent osimertinib, n (%)			
Yes	2 (3)	4 (7)	6 (5)
No	71 (97)	56 (93)	127 (95)
Immunotherapy within 6 mo of selpercatinib initiation, n (%)			
Yes	11 (15)	7 (12)	18 (14)
No	62 (85)	53 (88)	155 (86)

MAE, mucocutaneous AE.

a Other include cutaneous juvenile xanthogranulomatosis, rectal neuroendocrine cancer, infantile high-grade spindle cell sarcoma, and pancreatic adenocarcinoma.

### Incidence of MAEs and Impact on Therapy

**Figure 1:**
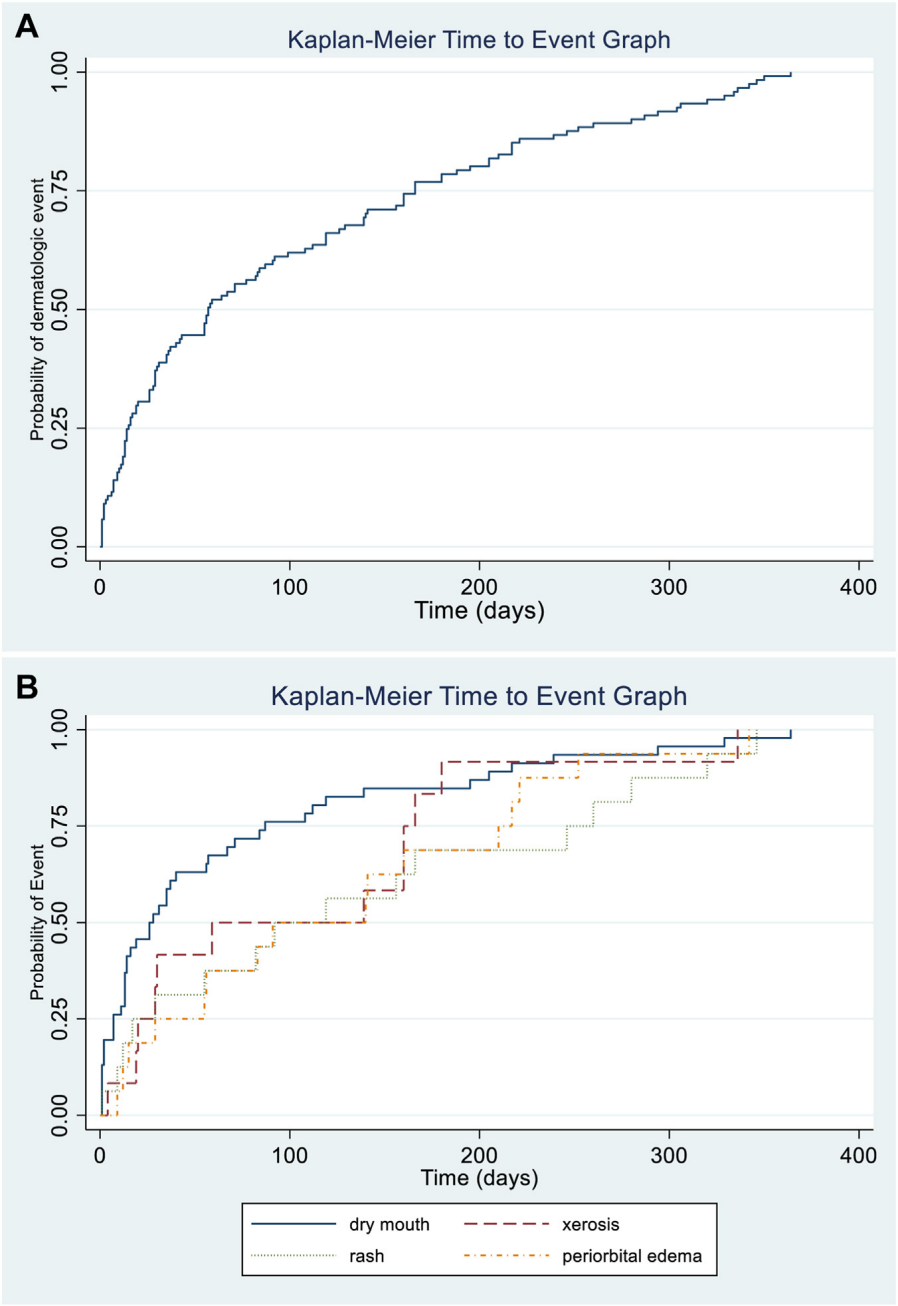
Time to onset of any grade selpercatinib-related mucocutaneous AE. (*A*) Illustrates time to onset of all-grade mucocutaneous AEs largely occurring within 100 days after initiation and increasing over time. (*B*) Shows time to onset of four common mucocutaneous AEs including dry mouth (xerostomia) with the earliest time to onset, followed by xerosis, periorbital edema, and then rash. AE, adverse event.

The yearly cumulative incidence of selpercatinib-related MAEs was 55% (95% confidence interval [CI]: 46–64), representing 73 of 133 patients with 126 reported MAEs. Of the 73 patients with MAEs, 38 (52%) had one unique MAE, including 20 patients (15%) with xerostomia alone. There were 21 (29%) patients with 2 MAEs, and 14 (19%) with more than two MAEs (Table 2), with xerostomia or rash (n = 11, 31%) and xerostomia or periorbital edema (n = 7, 20%) most often co-occurring. The median time to all-grade MAE was 57 (IQR: 15–166) days, with most MAEs occurring within the first 100 days after selpercatinib initiation (Fig. 1*A*). There were 18 (14%) patients who experienced an MAE-related interruption in therapy owing to rash, xerostomia, mucositis, or periorbital or facial edema (Table 3). Three (2%) patients were rechallenged with dose maintained, 10 (7%) with a 50% dose reduction, and five (4%) with a 25% dose reduction. One patient (1%) experienced mucositis when increasing their dosage from 80 mg to 160 mg twice daily. There were no reports of drug discontinuation.

**Table 2:** Mucocutaneous AEs, Clinical Characteristics, and Management

MAE Characteristics (N = 126)	Value, n (%)
Number of MAEs per patient (n = 73)	
1	38 (52)
2	21 (29)
3	10 (14)
4	4 (6)
Anatomical location of MAE	
Head/neck	79 (63)
Trunk	4 (3)
Extremities	17 (14)
Trunk and extremities	7 (6)
Head/neck and trunk	1 (1)
Head/neck and extremities	2 (2)
Total body	16 (13)
Time to MAE resolution, d	
Median (range)	68 (1–616)
Dermatology consultation	
Yes	16 (13)
No	110 (87)
Ophthalmology consultation	
Yes	6 (8)
No	120 (92)
Xerostomia	
OTC: saliva substitute oral solutions, lubricating rinses, gums, and xylitol/cellulose gum tablets	17 (14)
Pilocarpine and OTC lubricants	2 (2)
No treatment	30 (24)
Mucositis	
OTC: saliva substitute oral solutions, lubricating rinses, gums, and xylitol/cellulose gum tablets	1 (1)
Oral and topical steroids	1 (1)
Steroid rinse	4 (3)
No treatment	1 (1)
Rash	
Topical steroids ± ammonium lactate cream or oral antihistamines	12 (10)
Oral steroids ± topical steroids or oral antihistamines	5 (4)
Omalizumab ± topical steroids or oral antihistamines^a^	3 (2)
No treatment	6 (5)
Periorbital edema	
Topical steroids + caffeine compresses	3 (2)
Oral steroids + oral antihistamine	1 (1)
Cold/caffeine compresses	2 (2)
No treatment	10 (8)
Xerosis	
Emollient	4 (3)
Ammonium lactate cream	1 (1)
No treatment	7 (6)
Peripheral sensory neuropathy	
Opioid analgesics	1 (1)
No treatment	7 (6)
Pruritus	
Topical steroid	1 (1)
Emollient	1 (1)
No treatment	1 (1)
Infection	
Antibiotics or antiviral medication^b^	3 (2)
Nail changes (ridging, peeling)	
Poly-ureaurethane nail lacquer	1 (1)
No treatment	1 (1)

IgE, immunoglobulin E; MAE, mucocutaneous AE; OTC, over-the-counter.

a Omalizumab given in treatment-refractory cases of symptomatic grade 2 and 3 rashes with elevated IgE on laboratory evaluation.

b Included oral antibiotics for a case of cellulitis topical antibiotics for folliculitis, and antiviral medication for shingles.

**Table 3:** Clinical Presentation and Time to Onset of Selpercatinib-Related MAEs

MAE	Grade 1/2	Grade 3	All Grade	Median Time to Onset (IQR)	Drug Interruption/Dose Maintained, n (%)	Dose Reduction, n (%)
Rashes, n (CI)						
Maculopapular rash	9 (7)	1 (1)	10 (8)	74 (16–164)	0 (0)	3 (2)
Erythematous rash	4 (3)	0 (0)	4 (3)	183 (109–265)	1 (1)	1 (1)
Photosensitivity	5 (4)	0 (0)	5 (4)	129 (126–180)	1 (1)	1 (1)
Hives	1 (1)	0 (0)	1 (1)	1 (NA)	0 (0)	1 (1)
Palmar-plantar erythrodysesthesia syndrome	2 (2)	0 (0)	2 (2)	182 (98–266)	1 (1)	1 (1)
Exacerbation of chronic lesions^a^	2 (2)	0 (0)	2 (2)	133 (116–149)	0 (0)	0 (0)
Cutaneous, n (CI)						
Xerosis	12 (9)	0 (0)	12 (9)	59 (20–160)	0 (0)	0 (0)
Nail changes (ridging, peeling)	2 (2)	0 (0)	2 (2)	89 (73–104)	0 (0)	0 (0)
Edema, n (CI)						
Periorbital	16 (12)	0 (0)	16 (12)	91 (29–210)	0 (0)	2 (2)
Facial	2 (2)	0 (0)	2 (2)	182 (99–264)	0 (0)	1 (1)
Mucocutaneous, n (CI)						
Xerostomia	49 (37)	0 (0)	49 (37)	26 (7–87)	0 (0)	2 (2)
Mucositis	6 (5)	1 (1)	7 (5)	16 (6–188)	0 (0)	3 (2)
Sensory, n (CI)						
Peripheral neuropathy	8 (6)	0 (0)	8 (6)	77 (43–287)	0 (0)	0 (0)
Pruritus	3 (2)	0 (0)	3 (2)	55 (10–57)	0 (0)	0 (0)
Infectious, n (CI)						
Folliculitis, cellulitis or shingles	3 (2)	0 (0)	3 (2)	26 (15–31)	0 (0)	0 (0)
Total, n (%)	124 (98)^b^	2 (2)^b^	126 (100)^b^	57 (15–166)	3 (2)	15 (11)

CI, yearly cumulative incidence of the total population (N = 133); IQR, interquartile range; MAE, mucocutaneous AE; NA, not applicable.

a Includes psoriasis and actinic keratoses.

b Percentage based on 126 total MAE occurrences.

### Clinical Characteristics of MAEs

**Figure 2:**
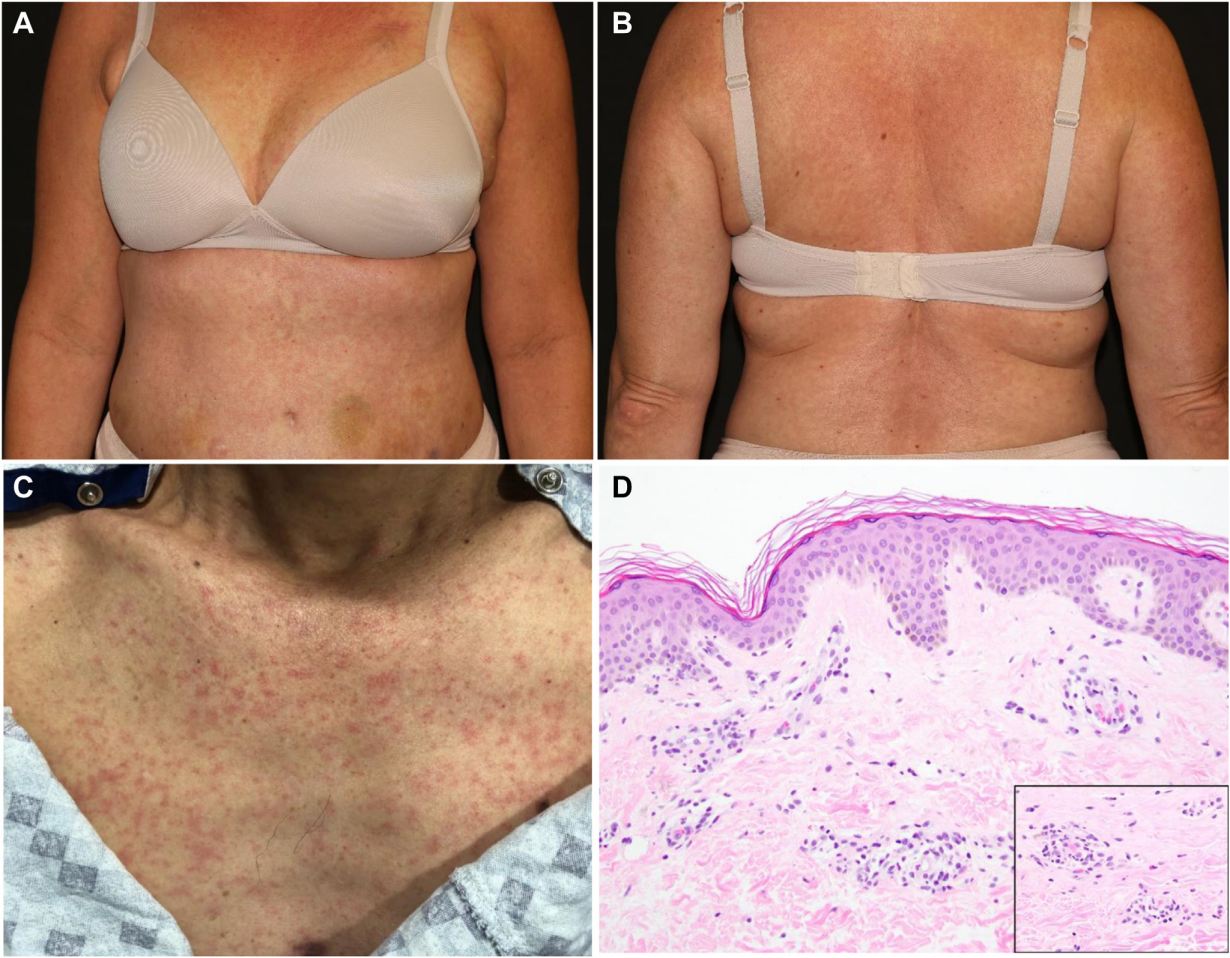
Selpercatinib-related maculopapular rash and associated histopathology. (*A*, *B*) Case 1: a 53-year-old female with a grade 2 generalized maculopapular rash occurring 12 days post selpercatinib initiation for NSCLC. The rash resolved 10 days later after therapy interruption and a 50 % dose reduction and treatment with oral and topical steroids and oral antihistamines. (*C*, *D*) Case 2: 56-year-old male with a grade 3 generalized maculopapular rash 9 days post selpercatinib initiation for NSCLC that required therapy interruption and a 50% dose reduction. The rash resolved 11 days later with the use of oral and topical steroids but reoccurred necessitating the use of omalizumab. (*D*) Histopathology from the skin biopsy of case 2: illustrates interface dermatitis with a perivascular lymphocytic infiltrate and scattered eosinophils (inset), compatible with a drug reaction. The image is 200× and the inset is 400×.

The most common MAEs included 37% (n = 49 of 126) with xerostomia, 18% (n = 24) with rash, 12% (n = 16) with periorbital edema, and 9% (n = 12) with xerosis (Table 3). The most common rash type included 8% (n = 10; 42% of all rashes) with a maculopapular rash (Fig. 2*A*–*D*), followed by 3% (n = 4) with erythematous rashes, 4% (n = 5) with photosensitivity, 2% (n = 2) with palmar-plantar erythrodysesthesia syndrome (PPE), 2% (n = 2) with exacerbation of underlying psoriasis or worsening actinic keratoses and 1% (n =1) with hives (Table 3). Almost all MAEs were grade 1 or 2 (n = 124, 98%).

MAEs noted to occur on average 30 days after initiation included xerostomia with median onsets of 26 (IQR: 7–87) and mucositis with 16 (IQR: 6–188) days after selpercatinib imitation (Fig. 1*B*). The median time to onset of most MAEs was less than 100 days after initiation, including pruritus (55 [IQR: 10–57] d), xerosis (59 [IQR: 20–160] d), peripheral neuropathy (77 [IQR: 43–287] d), periorbital edema (91 [IQR: 29–210] d), and rash (92 [IQR: 17–246] d). MAEs often affected the head or neck (n = 79, 63%), extremities (n = 17, 14%), and total body (n = 16, 13%) and resolved at a median of 68 (IQR: 14–142) days after onset. A total of 32 (25%) patients had a history of radiation therapy unrelated to the MAE. Of the 5% (n = 6) of patients on concomitant osimertinib therapy, two (3% of the total population) experienced an MAE. Of the 14% (n = 18) of patients who received immunotherapy within 6 months of selpercatinib initiation, 11 patients reported 19 MAE including dry mouth (n = 9), maculopapular rash (n 3), dry skin (n = 2), and mucositis (n = 2). Of these MAEs, 84% were grade 1, whereas the two grade 2 and one grade 3 MAE were maculopapular rashes.

The MAE group had more females than the non-MAE group (n = 38, 52% versus n = 26, 43%), were slightly younger (median age 62, range: 17–88 versus 64, range: 2–92 years old), and had more cases of NSCLC (n = 46, 63% versus n = 32, 53%), respectively (Table 1). However, none of these factors reached statistical significance: female sex (OR = 1.27, 95% CI: 0.77–2.63, *p* = 0.517), age (OR = 0.98, 95% CI: 0.96–1.01, *p* = 0.274), and NSCLC (OR 1.32, 95% CI: 0.57–3.04, *p* = 0.520).

### Management of MAEs

About half of MAEs did not receive treatment (n = 62, 49%), of these, 29% had documented self-resolution (n = 18). The other half of MAEs did require treatment (n = 64, 51%), of these, 61% had documented resolution after treatment intervention (n = 39). For those who underwent treatment for MAE, the time to resolution was shorter (mean days = 79) than those who were not treated (mean days = 242). Management was on the basis of the type of MAE (Table 2). Xerostomia was largely managed (n = 17, 14%) with over-the-counter saliva substitute solutions and lubricants; two (2%) required oral pilocarpine. Mucositis was mostly managed with steroid rinses (n = 4, 3%). Most rashes (n = 12, 10%) were treated with topical steroids plus or minus topical ammonium lactate and oral histamines. Five (4%) symptomatic grade 2 rashes required oral steroids, whereas three (2%) oral steroid-refractory symptomatic grade 2 and 3 rashes received omalizumab. Periorbital edema was managed with cold or caffeine compresses (n = 2, 2%), with three (3%) requiring the addition of topical steroids, and one (1%) oral steroids and antihistamines. Emollients were used to treat xerosis (n = 4, 3%) and pruritus (n = 1, 1%). There were 16 (13%) patients who required dermatology referral and six (8%) had an ophthalmology consult for persistent or severe MAE.

### Histopathology and Laboratory Assessment

Clinically indicated blood work was collected plus or minus 14 days from onset for 126 reported MAEs and was compared with 58 no-MAE patients’ routine laboratory values (two participants without MAEs did not have blood work within this range). Laboratory values did not differ on most complete blood counts with differential and comprehensive metabolic panel laboratory values other than percent lymphocytes, which was higher in the MAE (mean = 21.8, SD = 11.3, *p* = 0.005) compared with the no-MAE group (16.9, SD = 10.0). IgE levels were only collected in 17 patients (13%) with MAEs. The mean serum IgE value was 275 (SD = 294.5) kU/L, and eight of the 17 patients (47.0%) had serum IgE values higher than the accepted reference range of less than or equal to 214 kU/L. Of the eight patients with elevated IgE, MAEs included three patients with rash, three with facial or periorbital edema, one with skin infection, and one with xerosis. The difference between absolute eosinophils in those who had an MAE as compared with those without an MAE was not substantial. The one clinically indicated skin biopsy conducted for a maculopapular rash supported a drug reaction with an interface dermatitis with a perivascular lymphocytic infiltrate and eosinophils (Fig. 2*D*).

## Discussion

Here, we report a high yearly cumulative incidence of MAEs, often occurring within the first 100 days after selpercatinib initiation. This high incidence was owing to the inclusion of a comprehensive list of MAEs, such as the frequently-occurring xerostomia. Of note, one patient experienced an MAE while increasing their dose of selpercatinib. As preclinical animal studies found skin inflammation in cohorts of high-dose selpercatinib use, it is possible that MAEs may be dose-related.^8^ In the current study, MAE-related therapy interruptions necessitating dose reduction (11%) or discontinuation (0%) were less than the phase 1/2 LIBRETTO-001 trial of selpercatinib, which were 30% and 2%, respectively. This is likely owing to the clinical trial inclusion of therapy interruptions from all-cause AEs and not just MAEs.^2^^,^^9^ In a retrospective efficacy and safety analysis of 50 patients with *RET* fusion–positive NSCLC treated with selpercatinib, of the 20 patients (40%) who required dose reductions and 13 (26%) dose interruptions related to AEs, 12% of dose reductions and 8% of dose interruptions were attributable to xerostomia, peripheral edema, rash, and hypersensitivity reaction—comparable to the current study. They similarly had no reports of drug discontinuation, supporting the feasibility of avoiding therapy discontinuation while actively managing MAEs associated with selpercatinib.^10^

The most common MAEs included xerostomia, rash (predominantly maculopapular), periorbital or facial edema, and xerosis, virtually all of which were grade 1 or 2. Our reported incidences were similar to previous studies, with the LIBRETTO-001 trial reporting the most common MAEs being xerostomia (33%), edema (15%; including periorbital, eye, eyelid, face, and orbital edema), and rash (12%) which were maculopapular, erythematous, or pruritic (all of which were either grade 1 or 2).^2^^,^^9^ In a subset of 144 patients with *RET* fusion–positive NSCLC in this trial, the most common treatment-related AE was xerostomia (36%), and they had reports of xerosis (9%), both of which mirrored the current study.^2^ An additional study of patients with *RET* fusion–positive NSCLC treated with selpercatinib found fewer reports of xerostomia (26%), rash (8%), and xerosis (1%), and, in addition, reported peripheral edema (20%), hypersensitivity reaction (2%), and livedo reticularis (2%) as other MAEs.^10^ This myriad of related MAEs may be caused by various mechanisms of action, with consideration of RET kinase inhibition, haptogen formation, and possible off-target effects of selpercatinib.

The most reported MAE was xerostomia. Active salivation requires parasympathetic innervation of the otic and submandibular ganglia. Interestingly, Enomoto et al.^11^ determined that RET −/− mice had no sphenopalatine or otic ganglia, and fewer, smaller neurons in the submandibular ganglion. They further reported that Neurturin −/− mice (a ligand that plays a role in RET signaling) had normal-sized otic neurons, but fibers lacked proper maintenance and innervation.^11^^,^^12^ This suggests that disruption of RET signaling, as with RET tyrosine kinase inhibitor treatment, could impair the proliferation and maintenance of parasympathetic neurons in structures responsible for salivation. With further research, this may explain the high incidence of xerostomia and mucositis with the RET kinase inhibitor selpercatinib.

The second most common MAE was a rash that was largely maculopapular or erythematous. As selpercatinib is 97% bound to plasma proteins, it is possible that these exanthems reflect a delayed hypersensitivity reaction to haptens formed by selpercatinib-protein complexes, which often present with cutaneous exanthems.^4^^,^^13^

The third most common MAE was periorbital or facial edema. Common MAEs of vandetanib, a multikinase inhibitor used for the treatment of *RET-*altered cancers, include rash, acneiform dermatitis, xerosis, and photosensitivity, whereas MAEs of cabozantinib include PPE, stomatitis, oral pain, hair color changes, rash, and xerosis, with reports of mucositis.^14^ These are presumed to be because of various off-target effects. For example, scrotal erythema/edema and subungual splinter hemorrhages were hypothesized to be attributable to vascular endothelial growth factor (VEGF) inhibition causing vascular damage from mild trauma or friction.^15^ Of note, compared with older RET inhibitors like cabozantinib, the frequency of PPE is much lower and is all low-grade with selpercatinib.^16^

Finally, although selpercatinib largely inhibits wild-type and mutated RET kinase, inhibition of VEGF receptor (VEGFR) 1 and 3 is also observed.^4^ VEGFR-3 is largely expressed in the lymphatic epithelium and responsible for proper development and maintenance of lymphatic vasculature.^17^ Congenital *VEGFR3* mutations have been implicated in autosomal dominant primary congenital lymphoedema (Milroy disease), characterized by abnormal lymphatic drainage and chronic edema.^18^ In various mouse models inducing cutaneous inflammation, mice who received anti–VEGFR3 monoclonal antibodies had prolonged edema and inflammation, whereas VEGFR3 activation decreased inflammation.^19^^,^^20^ These findings suggest a possible role of VEGFR3 inhibition in selpercatinib-induced periorbital, facial, and peripheral edema and photosensitivity. Although these proposed mechanisms are compelling and may help explain these unique MAEs, there is a need for further research to explore and support these theories.

The time to onset of MAEs varied. Xerostomia and mucositis occurred early relative to other MAEs such as pruritus, xerosis, peripheral neuropathy, periorbital edema, and rash. In the phase 1/2 clinical trials of selpercatinib, hypersensitivity occurred at a median of 1.7 weeks (range: 6 d–1.5 y)—earlier than our findings.^21^ Whereas the time to onset of selpercatinib-related MAEs remains undefined in the literature, oral AEs from cabozantinib use are similarly reported to occur within 2 to 4 weeks after therapy initiation.^22^

Our analyses did not find any increased risk of MAE on the basis of sex, age, or cancer diagnosis. This is supported by two separate analyses of *RET*-altered NSCLC and thyroid cancers having similar reports of various MAEs.^2^^,^^9^

**Figure 3:**
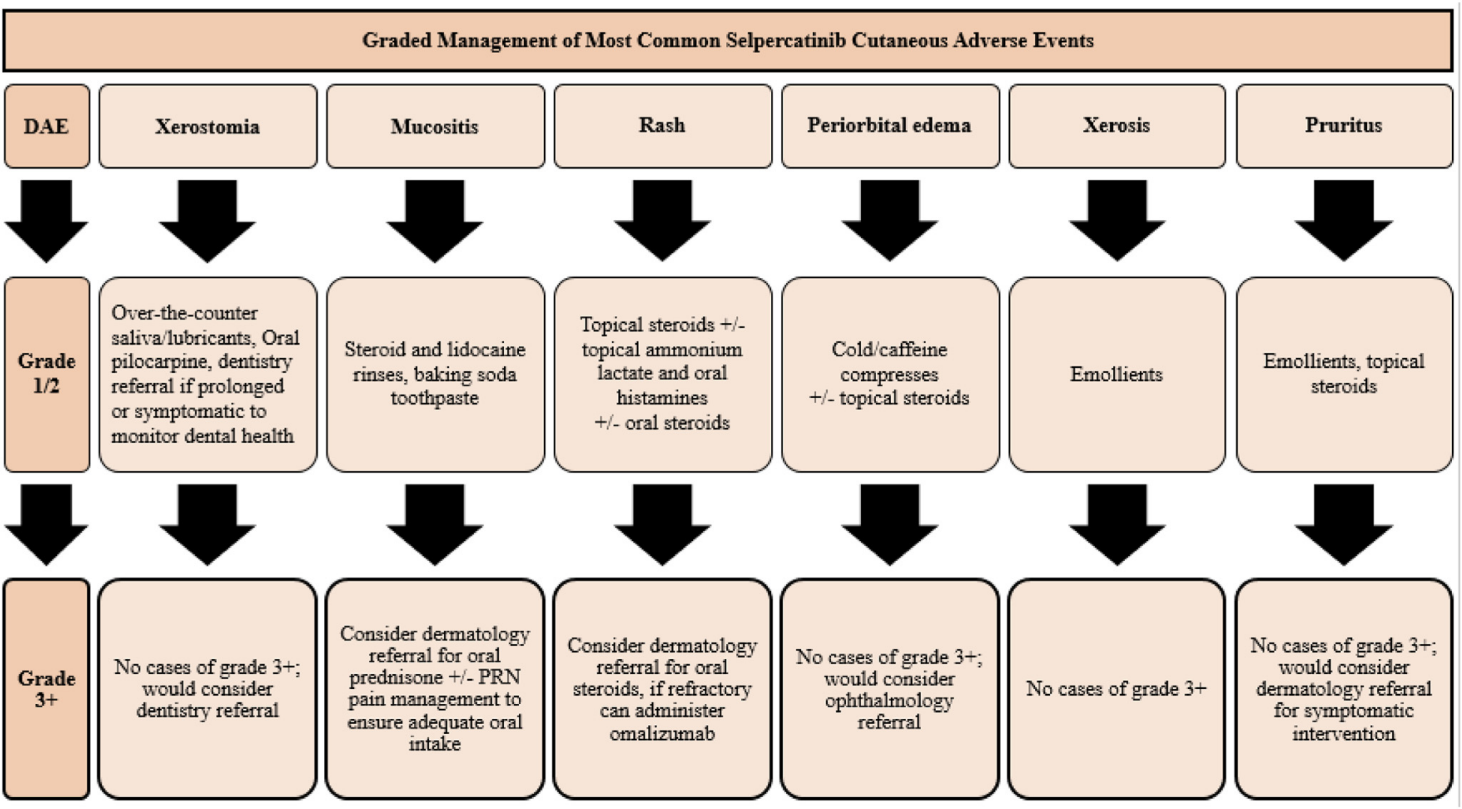
Management algorithm. DAE, dermatologic adverse event; PRN, as needed.

Management of MAEs was phenotype-based and can be visualized in Figure 3. Xerostomia was most often managed by over-the-counter saliva substitutes, lubricants, xylitol-based tablets and gums, and rarely, oral pilocarpine. Medication use is one of the most common causes of xerostomia, and intraoral topical agents are considered first-line.^23^ Saliva substitutes, lubricating rinses, and chewing gum stimulate saliva secretion and alleviate feelings of dry mouth in xerostomia from systemic disease, medications, and radiation.^24^^,^^25^ Use of xylitol tablets and gums has been found to prevent the incidence of dental carries.^26^ Finally, muscarinic agonists such as pilocarpine and cevimeline are efficacious in increasing salivation in states of pharmacology-induced and radiation-induced xerostomia, respectively.^27^^,^^28^ As such, we recommend the use of these management strategies. In cases in which xerostomia may be prolonged or remain symptomatic despite initial interventions, referral to dentistry may prove helpful in monitoring and management of gum disease, dental caries, and other sequelae of xerostomia.

Treated rashes were managed on the basis of severity, with most grade 1 and 2 rashes receiving topical steroids. For symptomatic grade 2 rashes, the addition of a course of oral steroids was used, and for treatment-refractory cases, a trial of omalizumab was initiated with the assistance of a supportive oncodermatology team. Available manufacturer labeling recommends holding selpercatinib and initiating oral steroids for hypersensitivity reactions. On resumption of therapy, labeling suggests continuation of steroids while initiating a reduced dose and increasing weekly by one dose level until the baseline dose is reached, followed by a steroid taper.^4^ Given that most drug interruptions occurred in those with rash, we recommend following this protocol and a dermatologic consult to allow for drug reinitiation without rash recurrence.

Periorbital edema was managed with cold or caffeine compresses and topical or oral steroids with or without antihistamines. Whereas there are no definitive treatments, periorbital edema does not cause harm to the eyes nor affect vision, and as such, drug discontinuation is not recommended. Instead, symptomatic relief by means of compresses, head elevation during sleep, and reassurance proved most effective in this cohort. Alternative therapies such as acetazolamide 500 mg, twice daily, and blepharoplasty are not advised owing to systemic adverse drug reactions and surgical risks, respectively, but may be used in extreme cases with input from Ophthalmology.

Emollients were used to help treat xerosis and pruritus (and are recommended). Given that selpercatinib is primarily metabolized by CYP3A4, strong consideration of concomitant CYP3A inhibitors should also be assessed to avoid possible dose-related toxicity.^8^

Histopathology supported a diagnosis of a drug reaction in the only skin biopsy conducted, which highlights how a dermatology consult may prove beneficial in complicated cutaneous eruptions of unknown cause. On the basis of the various MAEs, we recommend consulting with interdisciplinary teams including supportive oncodermatologists, ophthalmologists, and dentistry to minimize the symptomatic burden of MAEs.

Those with MAEs had a significantly higher percentage of lymphocytes compared with those without MAEs, possibly representative of an immunologic reaction. Lymphocytosis is predictive of an increase in immune-related AEs for patients receiving immunotherapy.^29^ As the only patients to have had IgE levels evaluated were those with MAEs, there was no group for comparison. Of those with this laboratory value, about half had an elevated IgE alongside rash or facial or periorbital edema supporting the use of this laboratory marker in detecting selpercatinib-related MAEs. Elevated IgE can be indicative of a type I hypersensitivity reaction; more importantly, high IgE can be therapeutically targeted with omalizumab.^30^ Future analyses may seek to compare IgE levels in those with and without selpercatinib-related MAEs to determine the significance.

Conclusions from this investigation are limited by its single-center retrospective design, the inclusion of patients on various dosages, the difference in dermatologic AE reporting between clinical trial and nonclinical trial settings, and the lack of matched placebo-treated controls. Furthermore, the incidence of peripheral and generalized edema was not explicitly assessed in this study. Given the high incidence of periocular edema, future directions may seek to characterize additional areas of edema that may be impacted by selpercatinib. Other future directions may include correlating MAEs with response to selpercatinib therapy.

The success of selpercatinib in phase 1/2 clinical trials has led to the initiation of phase 3 clinical trials, which will hopefully allow for expanded use of selpercatinib. Given that selpercatinib has been proven to prolong survival in those with *RET*-altered cancers, we expect long-term treatment and recommend close monitoring of associated MAEs. As such, identification and proper management of selpercatinib-related MAEs is of the utmost importance to encourage adherence to therapy, prevent dose interruptions, and maintain quality of life.

## CRediT Authorship Contribution Statement

**Rachel E. Reingold:** Conceptualization, Methodology, Investigation, Data curation, Formal analysis, Writing - original draft, Writing - review & editing, Visualization, Supervision.

**Rose Parisi:** Investigation, Writing - review & editing.

**Guilherme Harada:** Investigation, Writing - review & editing.

**Andrea P. Moy:** Investigation, Writing - review & editing.

**George Dranitsaris:** Data curation, Formal analysis, Writing - review & editing.

**Jasmine H. Francis:** Investigation, Writing - review & editing.

**Julia Canestraro:** Investigation, Writing - review & editing.

**Julia A. Lester:** Investigation, Writing - review & editing.

**Lauren A. Kaplanis:** Investigation, Writing - review & editing.

**Dazhi Liu:** Investigation, Writing - review & editing.

**Mario E. Lacouture:** Conceptualization, Methodology, Investigation, Data curation, Formal analysis, Writing - original draft, Writing - review & editing, Supervision.

**Alexander Drilon:** Conceptualization, Methodology, Investigation, Data curation, Formal analysis, Writing - original draft, Writing - review & editing, Supervision.

## Disclosure

Dr. Harada reports receiving honoraria from AstraZeneca, BeiGene, Bristol-Meyers Squibb, Daiichi, Eli Lilly, Johnson& and Johnson (J&J), Merck Sharp Dohme, Novartis, and Takeda. Dr. Drilon reports receiving honoraria from advisory board meetings of 14ner/Elevation Oncology, Amgen, Abbvie, ArcherDX, AstraZeneca, Beigene, BergenBio, Blueprint Medicines, Chugai Pharmaceutical, EcoR1, EMD Serono, Entos, Exelixis, Helsinn, Hengrui Therapeutics, Ignyta/Genentech/Roche, Janssen, Loxo/Bayer/Lilly, Merus, Monopteros, MonteRosa, Novartis, Nuvalent, Pfizer, Prelude, Repare RX, Takeda/Ariad/Millenium, Treeline Bio, TP Therapeutics, Tyra Biosciences, and Verastem; receives funds for associated research to institution from Foundation Medicine, Teva, Taiho, and GlaxoSmithKline; received equity from mBrace and Treeline; received fees for copyright of Selpercatinib-Osimertinib (pending); received royalties from Wolters Kluwer and UpToDate; received support for food/beverage from Boehringer Ingelheim, Merck, and Puma; and received CME honoraria from Answers in CME, Applied Pharmaceutical Science, Inc., AXIS, Clinical Care Options, EPG Health, Harborside Nexus, I3 Health, Imedex, Liberum, Medendi, Medscape, Med Learning, MJH Life Sciences, MORE Health, Ology, OncLive, Paradigm, Peerview Institute, PeerVoice, Physicians Education Resources, Remedica Ltd., Research to Practice, RV More, Targeted Oncology, TouchIME, and WebMD. Dr. Lacouture sits on the advisory board for J&J, Novocure, QED, Bicara, Janssen, Novartis, F. Hoffmann-La Roche AG, EMD Serono, AstraZeneca, Innovaderm, Deciphera, DFB, Azitra, Kintara, RBC/La Roche Posay, Trifecta, Varsona, Genentech, Loxo, Seattle Genetics, Lutris, OnQuality, Azitra, Roche, NCODA, Oncoderm, Takeda, and Apricity; and has contracted research with Lutris, Paxman, Novocure, J&J, US Biotest, OQL, Novartis, and AZ. The remaining authors declare no conflict of interest.
